# The leptospiral OmpA-like protein (Loa22) is a surface-exposed antigen that elicits bactericidal antibody against heterologous *Leptospira*

**DOI:** 10.1016/j.jvacx.2023.100382

**Published:** 2023-09-01

**Authors:** Edward J.A. Schuler, Dhara T. Patel, Richard T. Marconi

**Affiliations:** Department of Microbiology and Immunology, Virginia Commonwealth University Medical Center, 1112 E Clay St., Richmond, VA 23298, USA

**Keywords:** Leptospirosis, LIC10191, Loa22, LA0222, *Leptospira*

## Abstract

Leptospirosis is the most widespread zoonosis, affecting over 1 million humans each year, with more than 60,000 deaths worldwide. Leptospirosis poses a significant health threat to dogs, horses, cattle, and wildlife. The disease may be self-limiting or progress to a life-threatening multi-system disorder affecting the kidneys, liver, and lungs. Currently, bacterin vaccine formulations that consist of one or more laboratory-cultivated strains are used for prevention. However, the antibody response elicited by these vaccines is directed primarily at lipopolysaccharide and is generally serovar-specific. The development of broadly protective subunit vaccines for veterinary and human applications would be a significant step forward in efforts to combat this emerging and antigenically variable pathogen. This study assessed the properties and potential utility of the *Leptospira* Loa22 (*Leptospira* OmpA-like 22 kDa protein) protein as a vaccine antigen. Loa22 is a virulence factor that is predicted to transverse the outer membrane and present its N-terminal domain on the cell surface. This report demonstrates that diverse *Leptospira* strains express Loa22 *in vitro* and that the protein is antigenic during infection in dogs. Immunoblot and size exclusion chromatography revealed that Loa22 exists in monomeric and trimeric forms. Immunization of rats with recombinant Loa22 elicited bactericidal antibodies against diverse *Leptospira* strains. The immunodominant bactericidal epitopes were localized within the N-terminal domain using protein-blocking bactericidal assays. This study supports the utility of Loa22, or subfragments thereof, in developing a multivalent chimeric subunit vaccine to prevent leptospirosis and sheds new light on the cellular localization of Loa22.

## Introduction

Leptospirosis, which is caused by pathogenic *Leptospira* species, is the most common zoonosis worldwide. It has been reported on all continents except Antarctica [1]. The major maintenance hosts for *Leptospira* serovars vary but include rats, cattle, pigs, sheep, goats, dogs, and *peri*-domestic mammals. The *Leptospira* are invasive spirochetes that infect hosts by penetrating mucosal surfaces, conjunctival membranes, and skin abrasions [1]. In maintenance hosts, chronic infection is established in the kidneys, resulting in asymptomatic, perpetual shedding of spirochetes in urine. Transmission occurs through direct contact with infected urine, or via indirect contact with contaminated water or soil. The public health threat and economic burden of leptospirosis are well documented in both veterinary and human medicine [2], [3], [4], [5], [6]. The incidence of leptospirosis in dogs in the United States has been estimated to be ∼1400 cases per 10,000 canines [7]. In dogs, symptoms of leptospirosis may include fever, muscle ache, increased thirst, dehydration, vomiting, diarrhea, jaundice, or painful inflammation within the eyes. Severe leptospirosis can lead to kidney failure (with or without liver failure), lung disease, bleeding disorders and hemorrhage particularly on the gingiva and mucous membranes and death [8], [9]. In cattle, leptospirosis can cause estrous repetition, abortion, reduced milk production, and death. While the majority of livestock are vaccinated for leptospirosis, its economic impact on the livestock industry is staggering [10], [11].

Worldwide, 1.3 million cases are diagnosed in humans each year (95% CI 434,000–1,750,000) with approximately 60,000 deaths (95% CI 23,800–95,900) [12], [13]. The actual incidence is likely much higher due to incomplete laboratory testing, misdiagnosis, and the lack of rigorous reporting, particularly in patients that are not hospitalized. Human infections occur primarily through occupational, recreational, or avocational exposures. A recent report estimated the loss of productivity due to human infections to be between 29.3 and 52.3 billion dollars worldwide [14]. A study conducted in 2015 estimated that human leptospirosis results in 2.9 million Disability Adjusted Life years worldwide [15]. For comparison, this is over 70% of the global burden attributed to cholera. Most of the burden is due to premature death among young adults in poor, tropical countries. Torgerson et al. suggest that “these estimates place leptospirosis as the leading cause of disease burden amongst zoonotic pathogens” [15].

The family *Leptospiraceae* is comprised of at least 64 species, of which 38 are pathogenic and 26 are saprophytic [16]. Pathogenic isolates are divided into two subclades, designated as P1 and P2 [16]. At least twenty-four distinct *Leptospira* serogroups have been delineated based on lipopolysaccharide (LPS) serology [17]. These serogroups are further divided into over 300 serologically-distinct serovars [1], [18]. The antigenic diversity of leptospires has confounded efforts to develop broadly cross-protective vaccines and reliable diagnostic assays. All licensed and USDA-approved *Leptospira* vaccines are bacterin formulations consisting of cell lysates of one or more laboratory-cultivated serovars. *Leptospira* vaccines are widely used in dogs, horses, swine, cattle, and sheep. There is concern that the serovar-specificity of current vaccines will allow for the emergence of novel serovars [19]. It is unlikely that a traditional bacterin formulation can be generated that can achieve complete cross-protection. Hence, there is a pressing need to develop subunit vaccines with broad protective capability.

Development of broadly protective subunit vaccines for veterinary and human applications is a One Health priority. A recent review summarized different approaches and vaccine formulations for leptospirosis [20]. For the purpose of this study, we focus discussion on published studies that have sought to develop chimeric vaccine antigens. While several *Leptospira* antigens have been assessed as vaccine candidates, their ability to protect against diverse serovars has, in most cases, not been tested. A recombinant protein consisting of the variable regions of LigA and LigB, derived from *L. interrogans* serovar (sv.) Copenhageni strain (str.) Fiocruz-L130 conferred homologous protection in a lethal hamster model of acute leptospirosis, but not sterilizing immunity [21]. In another study, a LipL32, LigANI and LemA chimeric protein was delivered in a *Mycobacterium bovis* bacillus Calmette-Guérin formulation. Protection against lethal challenge with the homologous isolate, *L. interrogans* sv. Copenhageni str. Fiocruz L1-130, was demonstrated [22]. A chimeric recombinant protein, designated as r4R, was constructed based on computational predictions, and experimentally defined epitopes of OmpL1, LipL32 and LipL21 [23]. The chimera conferred protective and sterilizing immunity in guinea pigs against the homologous isolate, *L. interrogans* sv. Lai str. 56601.

Towards our goal of constructing a chimeritope (chimeric epitope-based protein) vaccinogen for leptospirosis, we have sought to identify immunodominant epitope-containing domains of *Leptospira* proteins. In a previous study we identified the immunodominant bactericidal epitopes of the General secretory pathway D (GspD) protein [24]. The goal of this study was to identify the antigenic determinants of the *Leptospira* virulence factor, Loa22 (*Leptospira* OmpA-like 22 kDa protein) [25] and assess its potential utility for inclusion in a multi-valent subunit vaccine. Loa22 has been reported to be essential for virulence [26].

Herein we demonstrate that Loa22 is expressed in culture by diverse strains and during natural infection in dogs. The N-terminal domain of Loa22 is surface exposed and the protein exists in both monomeric and trimeric forms. Hyperimmune serum generated in rats against recombinant Loa22 from *L. interrogans* sv. Copenhageni str. Fiocruz L1-130 displayed potent complement-dependent bactericidal activity against heterologous isolates of *Leptospira*. Using recombinant fragments of Loa22 in protein blocking bactericidal assays, the immunodominant bactericidal epitopes were localized within amino acid residues 27–97. The results of this study support consideration of Loa22, or fragments thereof, in the development of a multi-valent cross-protective, next-generation subunit vaccine to aid in the prevention of leptospirosis.

## Materials and methods

**Bacterial cultivation.***Leptospira* isolates (Table 1) were cultivated (30  °C) in EMJH medium supplemented with Probumin vaccine grade bovine serum albumin (BSA) (EMD Millipore) and 100 µg/mL 5-fluorouracil as previously described [27]. Growth was monitored by dark-field microscopy. Cells were recovered by centrifugation (10,000 rcf, 10 min, 4 °C) and washed twice with phosphate buffered saline (PBS; 137 mM NaCl, 2.7 mM KCl, 2 mM KH_2_PO_4_, 1 mM Na_2_HPO_4_; pH 7.4). Genomic DNA was isolated using the DNeasy® blood and tissue kit (QIAGEN) and DNA purity assessed by measuring the A_260_/A_280_ ratio (NanoDrop™ Spectrophotometer; Thermo Scientific).

**Table 1:** *Leptospira* isolates cultivated for this study.

Isolate	Source
*Leptospira interrogans* sv. Copenhageni str. Fiocruz L1-130	Human, Brazil
*Leptospira interrogans* sv. Canicola str. Kito	Canine, Brazil
*Leptospira interrogans* sv. Pomona str. LC82-25	Human, USA
*Leptospira noguchii* sv. Autumnalis str. Bonito	Human, Brazil
*Leptospira kirschneri* sv. Grippotyphosa str. RM52	Swine, USA
*Leptospira borgpetersenii* sv. Ballum str. M2	Mouse, Puerto Rico

**Dog serum samples.** Serum samples from client-owned dogs with symptoms consistent with leptospirosis (polyuria/polydipsia, dysuria, hematuria, abnormal liver and kidney values, acute renal failure, anorexia, vomiting, diarrhea and lethargy) were provided by Texas A&M University and Jolly Pond Veterinary Hospital (Williamsburg, VA) (Table 2). A subset of serum samples had been previously tested for antibodies to serovars Pomona, Icterohaemorrhagiae, Canicola, Grippotyphosa, Hardjo, Bratislava, Autumnalis, and Sejroe by microscopic agglutination test (MAT) [28], [29]. An agglutinating titer of 100 or greater against any serovar served as the threshold for a MAT positive score.

**Table 2:** Description of dog sera and antibody screening results.

		IgG Ab (ELISA)	
Serum identifier	MAT^a^	LipL32	Loa22
L0001	P/G/B/A	+	+
L0002	P/G/B/A	+	+
L0004	G/B/A	+	+
L0012^b^	–	–	–
L0021^b^	–	–	–
L0027	G	+	+
L0028	I/C/G/A	+	–
L0030	P/G/A	+	–
L0031	P/G/B/A	+	+
L0032	P/G/B/A	+	+
L0040^b^	–	–	–
L0055	P/G/B/A	+	+
L0058	P/I/G/A	+	–
L0060	P/I/C/G/B/A	+	–
JP-TD	NT^c^	+	+
JP-ARi	NT^c^	+	–
JP-BB	NT^c^	+	+
JP-MO	NT^c^	+	+
		100% (15/15)	66.7% (10/15)

a Antibody positive serovars as determined by MAT. Abbreviated as Pomona (P), Grippotyphosa (G), Autumnalis (A), Icterohaemorrhagiae (I), Canicola (C), and Bratislava (B) with additional details listed in Table 1. ^b^Negative control serum from healthy dogs (not included in percent cacluations). ^c^Not tested by MAT (NT).

**Generation of hyperimmune serum.** Antisera to recombinant proteins were generated in 8 week-old, female Sprague-Dawley rats (Charles River) using a 3-dose immunization protocol [30]. In brief, rats were immunized with 50 µg of protein (intraperitoneal; Freund’s complete adjuvant; Sigma-Aldrich) followed by two booster doses (25 µg protein; Freund’s incomplete adjuvant; Sigma-Aldrich), two weeks apart. One week after the final boost, the rats were euthanized by CO_2_ asphyxiation and cervical dislocation, and terminal bleeds conducted by cardiac puncture. Serum was harvested by centrifugation of whole blood in VACUETTE Z serum sep clot activator tubes (Greiner Bio-One). All animal studies were conducted in accordance with the Guide for the Care and Use of Laboratory Animals (Institute of Animal Research, National Research Council) with protocols approved by the Virginia Commonwealth University IACUC. Total IgG titers were determined as previously described [31].

**PCR and cloning.** PCR and cloning were performed as previously described [32]. Genes or gene fragments were PCR amplified from *L. interrogans* sv. Copenhageni str. Fiocruz L1-130 genomic DNA or plasmids carrying the target genes using Phusion® High-Fidelity DNA Polymerase (Thermo Scientific). Primers were designed with restriction sites for cloning (Table 3). Amplicons were analyzed by agarose gel electrophoresis and purified using the QIAquick PCR purification kit (QIAGEN). The amplicons were cut with the appropriate restriction enzymes (New England BioLabs), purified again, as above, and ligated into the multi-cloning site of pET-45b(+) (Novagen) using Instant Sticky-end Ligase Master Mix (New England BioLabs). Plasmids were propagated in *Escherichia coli* 5-alpha (DH5α) competent cells (New England BioLabs) and positive colonies identified by PCR using gene specific primers with GoTaq® Green Master Mix (Promega). PCR-positive colonies were inoculated into LB (Lennox) broth (Fisher Scientific) with ampicillin (100 mg/L), grown overnight (37 °C), and the plasmids purified as above. DNA sequences were determined on a fee-for-service basis (GeneWiz).

**Table 3:** Oligonucleotide primers for PCR and cloning.

Primer name	Sequence (5′ – 3′)^a^
loa22_27-197_-FWD	ATAGTCGGATCCCAAAGAGGAATCCGCAGCTCCTG
loa22_27-197_-REV	ATAGTCCGGCCGTTATTGTTGTGGTGCGGAAGTCG
loa22_27-97_-FWD	ATAGTCGGATCCC*TGGTGG*AAAGAGGAATCCGC
loa22_27-97_-REV	ATAGTCCGGCCGTTATGTTTTAGACCACTCGCTGAAATCACC
loa22_78-147_-FWD	ATAGTCGGATCCC*TGGTGG*GGATTTAGTTATAAAAAAGCGG
loa22_78-147_-REV	ATAGTCCGGCCGTTAAACTGCATTTGCACGAAGCTCAG
loa22_128-197_-FWD	ATAGTCGGATCCC*TGGTGG*CAAGCAGAAGGTG
loa22_128-197_-REV	ATAGTCCGGCCGTTATTGTTGTGGTGCGGAAGTCG
groEL-FWD	ATAGTCACCGGTATGGCGAAAGATATTGAATATAACG
groEL-REV	ATAGTCCGGCCGTTACATCATTCCGCCCATTCCTC
LipL32_22-272_-FWD	ATAGTCACCGGTGCTTTCGGTGGTCTGCCAAG
LipL32_22-272_-REV	ATAGTCCGGCCGTTACTTAGTCGCGTCAGAAGCAG
LipL41-FWD	ATAGTCGGATCCCGCTACAGTCGATGTAGAATATCCGG
LipL41-REV	ATAGTCCGGCCGTTACTTTGCGTTGCTTTCATCAAC

a Restriction site sequences incorporated into each primer are underlined. Two tryptophan codons (TGG), indicated by *italics*, were added after the restriction site on some primers.

**Induction and purification of recombinant proteins.** Protein expression and purification were performed as previously described [33]. In brief, plasmids carrying the gene or gene fragment of interest were transformed into *E. coli* BL21(DE3) cells (New England BioLabs) and protein expression induced with isopropyl β-D-1-thiogalactopyranoside (IPTG; 1 mM). Cells were recovered by centrifugation and suspended in binding buffer (500 mM NaCl, 20 mM Na_2_HPO_4_, 20 mM imidazole; pH 7.4) supplemented with 1% protease inhibitor cocktail (Sigma-Aldrich) and 2.5 U/mL Pierce™ Universal Nuclease (Thermo Scientific). Cells were lysed using an EmulsiFlex-C3 high-pressure homogenizer (3 passes, 100-150 MPa, 4 °C). The soluble and insoluble fractions of the homogenate were separated by centrifugation and assessed by SDS-PAGE. Proteins that fractionated with the soluble phase were purified by nickel affinity chromatography on an ÄKTA Pure 25 M (Cytiva) FPLC platform using 1 mL HisTrap FF columns (Cytiva) [34]. Protein-containing eluates were pooled and dialyzed into PBS. Proteins that fractionated with the insoluble phase were purified by gravity flow using nickel-charged Poly-Prep Chromatography Columns (Bio-Rad) with a 1 mL His-Bind Resin bed (Millipore). In brief, cell pellets were resuspended in urea binding buffer (8 M urea, 300 mM NaCl, 50 mM NaH_2_PO_4_, 10 mM imidazole, 1 mM THP; pH 8.0; 30 min, RT; with frequent mixing) supplemented with 1% protease inhibitor cocktail (v/v) and 2.5 U/mL Pierce™ Universal Nuclease. Cellular debris was removed by centrifugation and the supernatant collected and loaded onto the column. The column was washed with urea binding buffer and protein eluted with urea elution buffer (8 M urea, 500 mM NaCl, 250 mM imidazole, 20 mM NaH_2_PO_4_; pH 8.0). The eluate was dialyzed sequentially against buffer A (4 M urea, 20 mM ethanolamine, pH 11.7), buffer B (2 M urea, 20 mM ethanolamine, 2 mM cystine, pH 11.7), buffer C (20 mM Tris Base, 2 mM cysteine, 0.2 mM cystine, pH 10.7), and PBS at 4 °C (at least 4 h per step) [33]. Protein concentrations were determined by bicinchoninic acid (BCA) assay (Pierce™) and purity assessed by sodium dodecyl sulfate–polyacrylamide gel electrophoresis (SDS-PAGE).

**SDS-PAGE and immunoblot analyses.** Recombinant Loa22 (500 ng) or *Leptospira* cells (3.33 OD_600_/mL) were diluted in SDS-PAGE buffer (20% glycerol (v/v), 4% SDS (w/v), 2% β-ME (v/v), 0.1% bromophenol blue (w/v), 250 mM Tris; pH 6.8), sonicated and heated (10 min; 99 °C). Samples were separated in Criterion™ TGX™ AnykD gels (Bio-Rad) with Tris/Glycine/SDS Buffer (Bio-Rad) and stained with Coomassie brilliant blue R-250 (Thermo Scientific). Precision Plus Protein Dual Color Standard (Bio-Rad) served as a molecular weight (MW) marker. Stained proteins were visualized and imaged on a ChemiDoc™ Touch imaging system (Bio-Rad) using auto-optimal settings. Proteins were transferred to polyvinylidene difluoride (PVDF) membranes using the Trans-Blot® Turbo™ system (Bio-Rad; high MW preset). The membranes were immersed in blocking solution (BKS; 5% non-fat dried milk, 0.2% Tween-20, in PBS). Primary Ab was added (1:1000 in BKS; 1 hr), decanted, the membranes washed three times (0.2% Tween-20 in PBS), and goat anti-rat IgG HRP (Novus Biologicals) secondary Ab added (1:40000 in BKS; 1 hr). After washing, Clarity Western ECL Substrate (Bio-Rad) was added (5 min) and images were captured as above.

**Indirect enzyme-linked immunosorbent assay.** Indirect ELISA analyses were performed as previously described [35]. In brief, LipL32, Loa22, or FhbB (500 ng/well) were coated on 96-well plates (Corning) in triplicate in bicarbonate buffer (35 mM sodium bicarbonate, 15 mM sodium carbonate; pH 9.6; 100 μL/well; overnight, 4 °C). Recombinant *Treponema denticola* 35405 FhbB [36] served as a negative control. Primary Ab was utilized at a 1:1000 dilution, and rabbit anti-canine IgG horseradish peroxidase (Novus Biologicals) at a 1:15000 dilution in BKS (1 hr each), both followed by six washes (0.5% Tween-20 in PBS). Chromogenic solution (10 mM citric acid, 0.8 mM 2,2′-azino-bis(3-ethylbenzothiazoline-6-sulfonic acid (ABTS), 0.003% H_2_O_2_ (Sigma-Aldrich)) was added, the plates incubated in the dark (30 min), and A_405nm_ measured using an ELx808 plate reader (BioTek). Samples with an absorbance value at least two-fold greater than that of the negative control protein, FhbB, were scored as positive.

**Size exclusion chromatography (SEC).** SEC was performed as previously described, with some modifications [37], using a TSKgel G4000SW_XL_ column (Tosoh Bioscience) attached to a 1260 Infinity II HPLC system (Agilent), and PBS as the mobile phase. To generate a standard curve, a 15–600 kDa standard protein mix (Millipore Sigma) was prepared as per manufacturer instructions, filtered through a 0.22 µm PVDF membrane, injected into the column (20 μL) and eluted using PBS at a flow rate of 1.0 mL/min. Retention time (Rt; min) was plotted versus Log(MW) to yield a standard curve with formula: log(MW) = -0.3506*(Rt) + 4.875 with R^2^ = 0.9870. To assess the oligomeric state of Loa22_27-197_, recombinant protein (50 µg; PBS) was filtered, injected into the column (25 μL) and eluted with PBS as above. Fractions were collected based on mAU and assessed by SDS-PAGE and immunoblotting using anti-Loa22_27-197_ antiserum.

**Immunofluorescence assay.***L. interrogans* sv. Copenhageni str. Fiocruz L1-130 cells (mid-log phase) were harvested by centrifugation (2,000 rcf, 10 min), washed twice with PBS (with 5 mM MgCl_2_) and resuspended in PBS (10^8^ cells/mL). Five µL aliquots of the cell suspension were spotted onto Superfrost™ Plus Microscope Slides (Fisher Scientific) and air dried (1 hr). Cells on one set of slides were permeabilized by immersion in acetone (10 min) and then dried. Blocking solution (3% BSA, 0.2% Tween-20, in PBS) was applied to slides for 30 min. Hyperimmune serum (1:1000 in blocking solution) was then spotted onto the slides (30 min, RT), followed by a wash (3 times; 0.2% Tween-20 in PBS). Goat anti-rat IgG Alexa Fluor™ 568 (Invitrogen) secondary Ab (1:500 in blocking solution) was applied (in the dark; 30 min). Slides were washed as above, and ProLong™ Gold Antifade Mountant with DAPI (Invitrogen) was added, and a cover slip applied. Fluorescence was visualized on an Olympus BX53 dark field microscope using an X-Cite® series 120 Q fluorescence lamp and images captured with an Olympus DP74 camera.

**Bactericidal antibody assays.** To ensure that active complement levels were similar in all assays, endogenous complement present in the hyperimmune sera was heat inactivated (HI) (56 °C, 30 min). For some control assays, the normal human serum (NHS; COmpTech) exogenous complement source was also HI. The bactericidal activity assays were conducted by incubating 20% (v/v) of a mid-log phase culture with 20% (v/v) HI anti-Loa22_27-197_, 20% HI anti-LipL32, or 20% HI anti-GroEL antiserum, 20% (v/v) NHS or HI-NHS and 40% (v/v) EMJH in 20 μL final volume for 16 hr at 30 °C. HI pre-immune (PI) rat serum with NHS or HI-NHS added served as negative bactericidal activity controls. The average number of live cells in five fields of view was determined by visual counting using wet-mounts and dark field microscopy. Percent killing, relative to the average number of live cells treated with rat pre-immune serum, was determined for each treatment. Further, the bactericidal activity of anti-Loa22_27-197_ against heterologous strains was measured as above. To identify the domain(s) of Loa22_27-197_ that elicit bactericidal antibody, an antibody-blocking bactericidal assay was employed. Anti-Loa22_27-197_ antiserum was pre-incubated with recombinant Loa22_27-197_, Loa22_27-97_, Loa22_78-197_, or Loa22_128-197_ (250 ng/µL antiserum) for 16 hr at 30 °C. The antiserum-protein mixtures were then used to set up bactericidal assay reactions, as detailed above. Anti-Loa22_27-197_ antiserum incubated with *T. denticola* FhbB, or with no protein added, served as controls. Percent cell killing was determined as above. All bactericidal assays were performed in triplicate with three technical replicates.

**Fig. 1:**
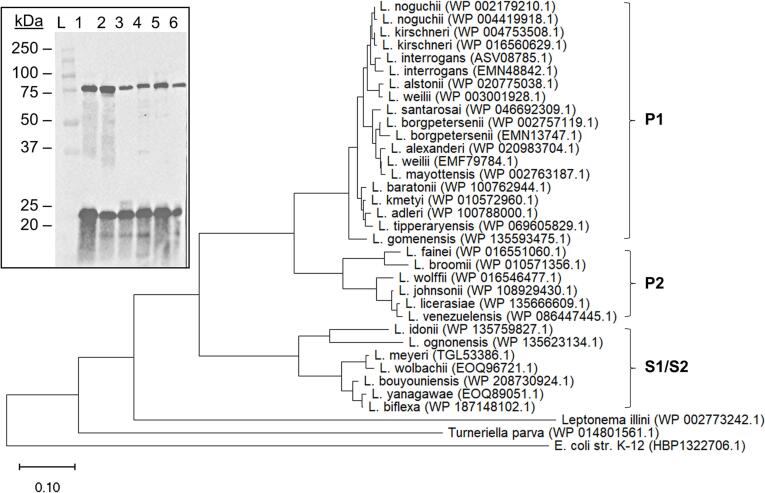
Loa22 neighbor-joining tree and *in vitro* expression among diverse Leptospira. Loa22 amino acid sequences were retrieved from NCBI (accession numbers indicated), aligned with ClustalW (default settings) [38], and a phylogenetic neighbor-joining tree constructed in MegaX [39]. Clusters corresponding to pathogenic (P1 and P2) and saprophytic (S1/S2) subclades [16] are indicated. The insert is an immunoblot in which whole cell lysates of: 1) L. interrogans sv. Copenhageni str. Fiocruz L1-130, 2) L. kirschneri sv. Grippotyphosa str. RM52, 3) L. noguchii sv. Autumnalis str. Bonito, 4) L. interrogans sv. Pomonna str. LC82-25, 5) L. interrogans sv. Canicola str. Kito, and 6) L. borgpetersenii sv. Ballum str. M2 were screened with anti-Loa22_27-197_ antiserum, as indicated in the text. The migration position of molecular weight markers is shown on the left.

**Bioinformatic and statistical analyses.** Loa22 amino acid sequences were retrieved from NCBI (accession numbers indicated in Fig. 1), aligned with ClustalW (default settings) [38], and a phylogenetic neighbor-joining tree constructed in MegaX [39]. Putative B cell epitopes were identified using Bepipred-2.0 [40]. Statistical analyses were conducted using GraphPad Prism version 9.2.0 for Windows, GraphPad Software, San Diego, California USA, https://www.graphpad.com. Ordinary one-way ANOVAs were performed assuming Gaussian distribution of residuals and significance (*P* <.05) calculated from comparison of the means of each group. Ordinary two-way ANOVAs were fit with a full interaction model and significance (*P* <.05) calculated by Tukey’s multiple comparisons test using default settings. Student’s *t*-tests were performed in Microsoft Excel with homoscedastic, two-tailed distribution.

## Results

**Fig. 2:**
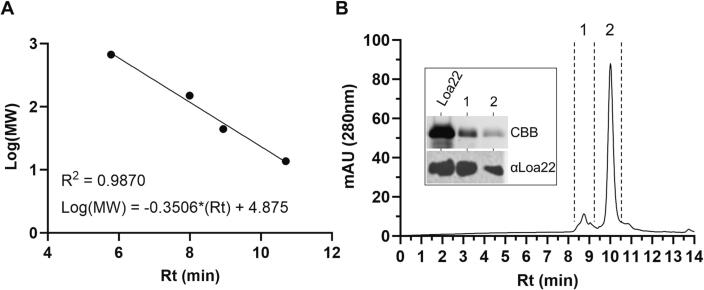
Loa22 exists as a monomer and trimer. The oligomeric state of Loa22_27-197_ was assessed by size-exclusion chromatography (SEC). A standard curve was generated (Panel A) using globular protein standards. The log of the MW of the standards was plotted against retention time (Rt) to calculate the shown linear regression formula. Panel B shows the Loa22_27-197_ SEC chromatogram. The eluate under peaks 1 and 2 was collected (fractions denoted by dashed lines), analyzed by SDS-PAGE, and screened by immunoblot with anti-Loa22_27-197_ antiserum. CBB: coomassie brilliant blue.

**Loa22 is conserved among *Leptospira* isolates and exists in monomeric and trimeric forms.** The BLASTp analyses using WP_000239305.1 as the query detected Loa22 homologs in both pathogenic and saprophytic *Leptospira* spp. The clades defined by Loa22 sequences are consistent with the division between pathogenic and saprophytic clades (Fig. 1), as well as the separation of the pathogenic P1 and P2 clades. The percent amino acid (AA) identity of Loa22 sequences among pathogenic *Leptospira* species ranged from 90.2% to 100% [16]. Immunoblot analyses of *Leptospira* whole cell lysates revealed that Loa22 is expressed by all strains tested and exists as both a monomer and trimer (Fig. 1, insert). For SEC, a standard curve was generated (Fig. 2 A). Loa22_27-197_ was assessed by SEC. The calculated MW of the protein under peaks 1 (66.3 kDa) and 2 (23.0 kDa) were consistent with a trimer and monomer, respectively. Eluate under peaks 1 and 2 was collected for immunoblot analyses using anti-Loa22_27-197_ antiserum (Fig. 2 B). Measurement of the area under each peak revealed that a majority of the recombinant protein exists as a monomer (89% of the total).

**Fig. 3:**
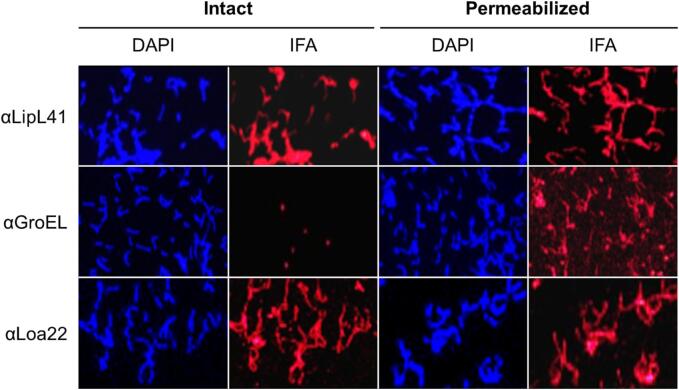
Loa22 is surface exposed. *L. interrogans* str. Fiocruz L1-130 cells were spotted onto slides and screened with each antiserum indicated to the left. Images from intact cells (air dried) and permeabilized (acetone fixed) cells are shown. Antibody binding was detected using goat anti-rat IgG AlexaFluor^TM^ 568. Cells were stained with DAPI as a control. IFA: immunofluorescence assay. DAPI: 4′,6-diamidino-2-phenylindole.

**Loa22 is surface exposed and antigenic.** The surface exposure of Loa22 on *in vitro* cultivated *L. interrogans* sv. Copenhageni str. Fiocruz L1-130 cells was assessed by immunofluorescence assay (IFA) (Fig. 3). Sera generated against LipL41 and GroEL were used as controls. LipL41 [41] and GroEL [42] are known surface exposed and intracellular proteins, respectively. As expected, LipL41 was detected in both intact and permeabilized cells, whereas GroEL was detected exclusively in permeabilized cells. Loa22 was detected in both the intact and permeabilized cells indicating that at least a portion of Loa22 is surface exposed. Expression of *loa22 in vivo* was indirectly assessed by screening for anti-Loa22 antibodies in the serum of dogs diagnosed with leptospirosis (n = 15 MAT(+); n = 3 MAT(-)) (Table 2). As expected, all MAT(+) dogs were positive for LipL32 antibodies and negative for FhbB antibodies. Ten of the 15 LipL32 antibody positive dogs were antibody positive for Loa22. All three MAT(-) dogs were negative for every antigen. These data demonstrate that Loa22 is expressed and antigenic in a majority of the client-owned dogs with natural leptospirosis.

**Fig. 4:**
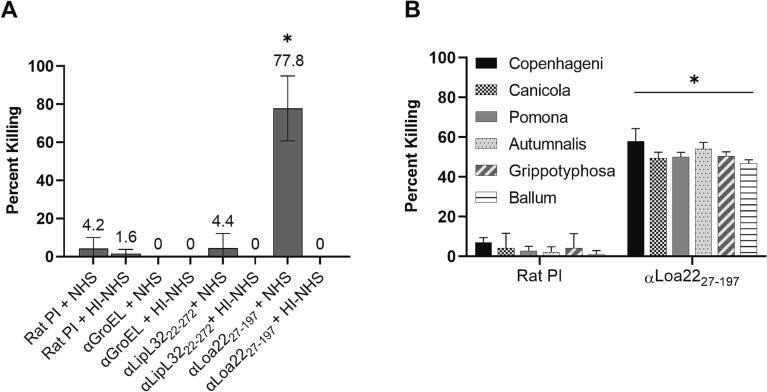
Bactericidal activity of anti-Loa22 antiserum against heterologous *Leptospira* spp. In panel A, mid-log phase *L. interrogans* str. Fiocruz L1-130 cells were incubated with anti-LipL32_22-272_, anti-GroEL, or anti-Loa22_27-197_ antiserum, or pre-immune serum (PI) in the presence of complement certified normal human serum (NHS) or heat-inactivated NHS (HI-NHS). All assays were done in triplicate and the average percent killing calculated from one experimental replicate shown above each bar. In panel B, anti-Loa22 antiserum was assessed for bactericidal activity against heterologous *Leptospira* isolates with NHS or HI-NHS. The serovars tested are indicated in the key. Significance (* *P* <.05) was determined by one-way ANOVA in GraphPad Prism and Student’s *t*-test in Microsoft Excel as described in the text.

**Anti-Loa22_27-197_ antiserum has complement-dependent bactericidal activity against diverse *Leptospira* strains*.*** The potential bactericidal activity of anti-Loa22_27-197_ antiserum was assessed by incubation with *L. interrogans* sv. Copenhageni str. Fiocruz L1-130 cells with and without NHS or HI-NHS. Significant bactericidal activity was observed with antiserum in the presence of NHS (77.8% killing; *P* <.05) (Fig. 4 A). No killing was observed with antiserum plus HI-NHS or with cells incubated with PI serum (PI) and NHS. Anti-LipL32_22-272_ [43] and anti-GroEL [42] antisera served as negative controls, and, as expected, were not bactericidal. It can be concluded that the bactericidal activity of anti-Loa22_22-197_ antiserum occurs through an antibody-mediated, complement-dependent mechanism. The potential bactericidal activity of heterologous strains by anti-Loa22_27-197_ antibodies (in the presence of NHS or HI-NHS) was also assessed (Fig. 4 B). Significant killing was observed across all isolates tested (Fig. 4 B). The isolates tested covered 6 distinct serovars and 4 species. These results suggest that Loa22 may be of utility in a multi-valent subunit vaccine to confer cross-protection.

**Fig. 5:**
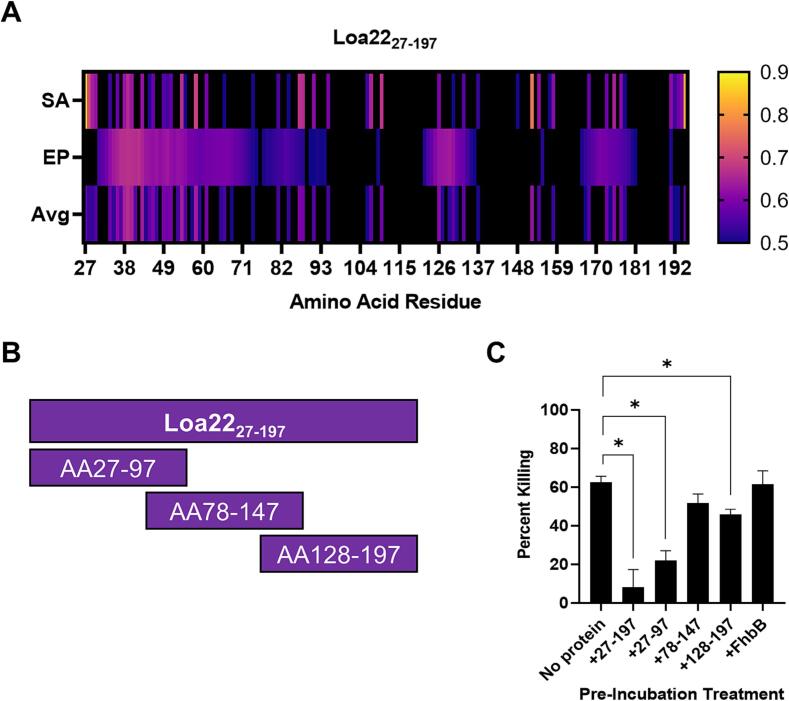
Localization of Loa22 immunodominant bactericidal epitopes. BepiPred-2.0 [40] B cell epitope predictions are shown (Panel A). Solvent accessibility (SA) and B cell epitope probability (EP) values (0.5–1.0) for each residue are presented as a heatmap along with the average (Avg) of SA and EP. Probabilities less than 50% (0.5) were excluded (black). In panel B, a schematic of the Loa22 subfragments used to localize the immunodominant bactericidal epitopes is shown. The segment of the protein included in each fragment (amino acids: AA) is indicated within each box. The putative signal sequence was excluded. Results of antibody blocking bactericidal assays are presented in panel C. Anti-Loa22_27-197_ antiserum was pre-incubated before being mixed with cells with the Loa22_27-197_, Loa22 fragments (as indicated in the figure) or an irrelevant protein (FhbB; negative control). Percent cell killing was measured as detailed in the text. Significance (* P <.05) was determined by one-way ANOVA using GraphPad Prism.

**Localization of the bactericidal epitope(s) of Loa22.** Several approaches were employed to localize the immunodominant bactericidal epitopes of Loa22. Predictions obtained using Bepipred-2.0 [40] revealed potential clustering of B cell epitopes within the N-terminal 97 residues (Fig. 5 A). Based on these predictions Loa22_27-197_ and Loa22 fragments (Loa22_27-97_, Loa22_78-147_, and Loa22_128-197_) were produced. The fragments were of similar length and contained 20 amino acid overlaps (Fig. 5 B). To identify the domain(s) of Loa22 that elicit bactericidal antibodies, anti-Loa22_27-197_ antiserum was preincubated with Loa22_27-197_ (positive antibody blocking control), Loa22_27-97_, Loa22_78-147_, Loa22_128-197_ or FhbB (negative antibody blocking control) (Fig. 5 C). The rationale being that fragments that contain bactericidal epitopes will be bound by the bactericidal antibodies resulting in reduced killing. The serum-protein mixtures were incubated with *L. interrogans* sv. Copenhageni str. Fiocruz L1-130 cells with and without NHS or HI-NHS. Untreated anti-Loa22_27-197_ antiserum (no protein added) served as the positive control for bactericidal activity. A significant reduction in killing was observed in the Loa22_27-197_ control reaction, and with two of the three Loa22 subfragments: Loa22_27-97_ and Loa22_128-197_ (*P* <.05); however, Loa22_27-97_ more effectively (*P* <.05) blocked bactericidal antibody compared to Loa22_128-197_ (Fig. 5 C). Pre-incubation of the serum with *T. denticola* FhbB (an irrelevant protein) had no effect on bactericidal activity. The data indicate that bactericidal epitopes are concentrated between amino acid residues 27 and 97.

## Discussion

Achieving broad, cross-protective immunity remains a significant challenge in the development of vaccines for leptospirosis. Conserved surface-exposed virulence factors are attractive candidates due to the of antibody to act synergistically by blocking the function of the virulence factors and eliciting broadly-protective antibodies [44], [45]. This study is focused on the *Leptospira* Loa22 protein. Several functional roles have been proposed for Loa22, including evasion of innate immunity and binding of host extracellular matrix components [26], [46], [47], [48], [49], [50]. *Himar1* transposon mutagenesis and complementation provided direct evidence that *loa22* is essential for infection in guinea pig and hamster models [26]. The potential utility of Loa22 as a vaccinogen has not received significant attention. In this study, we characterized the antibody response to Loa22 to examine its potential utility as a component of a multi-valent subunit vaccine for leptospirosis.

Studies that have assessed the expression of Loa22 have not, to our knowledge, confirmed or compared expression levels amongst strains. This remains a shortcoming of our understanding of its role across diverse isolates. In this report, Loa22 production during cultivation was detected in diverse strains using hyperimmune serum raised against Loa22_27-197_ (LIC10191). This cross-reactivity of the antiserum is consistent with Loa22 sequence conservation. Loa22 production by *Leptospira* grown under host-like conditions or during infection in different mammals has been investigated in other studies. Eshghi et al. reported an increase in Loa22 production by *Leptospira interrogans* sv. Copenhageni when cultivated under host-like conditions in media containing fetal bovine serum and limited iron [51]. Nally demonstrated upregulation of *loa22* in *L. interrogans* serogroup Icterohaemorrhagiae str. RJ19115 freshly isolated from urine and then cultivated in dialysis membrane chambers implanted in the peritoneal cavity of rats [52]. Evidence for expression during human infection comes from the detection of Loa22 antibodies in patient sera [53], [51]. As we report here, it is noteworthy that while Loa22 is surface exposed, as demonstrated by IFA, and genetically and antigenically conserved among pathogenic *Leptospira* species, antibodies to Loa22 were not detected in all dogs diagnosed with leptospirosis. This includes dogs that were antibody positive for LipL32 and positive by the MAT for multiple serovars. As discussed below, this observation has implications for its potential use in vaccines and diagnostic assays.

Immunoblot analyses of cultivated *Leptospira* strains and SEC analyses of recombinant Loa22 revealed that it exists as a monomer and as a trimer. Notably, the trimer proved highly stable as it was resistant to denaturing conditions and boiling. The N-terminal domain of Loa22 may facilitate the formation of a non-covalently linked trimeric complex, possibly due to interactions between the hydrophobic faces of amphipathic alpha helices [54]. The formation of a trimer is consistent with a potential porin function as inferred from conserved domain analyses of the Loa22 sequence. The molecular basis for the formation of a highly stable trimer will require further research.

Immunization of rats with Loa22 elicited a high-titer IgG response. The antibodies were bactericidal and killed homologous and heterologous strains. Bactericidal activity was strictly antibody-mediated and complement-dependent, as no killing was observed when incubated with antibody alone or with antibody and HI-complement certified NHS. Using recombinant overlapping Loa22 fragments, the region of Loa22 that stimulates production of bactericidal antibodies was localized within amino acids 27 to 97. This same region has several amino acid segments that are predicted with moderate to high probability to be solvent accessible and to harbor B cell epitopes. The identification of bactericidal epitope(s) within Loa22 is important as it will allow for the incorporation of immune-relevant regions in a multivalent chimeric subunit vaccine.

Collectively, the results presented within suggest that a single Loa22 protein vaccine formulation would not in and of itself provide broad or full protection. The percent killing measured in the bactericidal assays was not complete, ranging from 50 to 80%. In addition, we did not detect anti-Loa22 antibodies in all dogs that were either positive by MAT or for anti-LipL32 antibody. It is our hypothesis, based on the analyses presented here and earlier literature, that recombinant protein(s) comprised of bactericidal epitopes from multiple different *Leptospira* proteins will be required to elicit broad protection. Previous work with the Lyme disease spirochete, *Borreliella burgdorferi*, demonstrated proof of concept for the use and efficacy of chimeritopes [55], [33], [30]. Vanguard® crLyme (Zoetis) is a USDA-approved and commercially available multi-protein subunit vaccine for Lyme disease prevention in dogs [56], [57]. The chimeritope component of Vanguard® crLyme is a novel recombinant protein (Ch14) consisting of linear epitopes derived from multiple OspC variants. A similar chimeritope based protein may allow for circumvention of the well-documented diversity of the *Leptospira* species, strains and serovars.

## Declaration of Competing Interest

The authors declare that they have no known competing financial interests or personal relationships that could have appeared to influence the work reported in this paper.

## Data Availability

Data will be made available on request.
